# A Caregiver Perspective for Partners of PTSD Survivors: Understanding the Experiences of Partners

**DOI:** 10.3390/bs14080644

**Published:** 2024-07-26

**Authors:** Christopher J. Cannon, Matt J. Gray

**Affiliations:** Department of Psychology, University of Wyoming, Laramie, WY 82072, USA

**Keywords:** PTSD, couples, social support, intimate partner, caregiving

## Abstract

Research affirms that survivors of post-traumatic stress disorder (PTSD) experience psychological distress that affects their romantic partners, and that a bi-directional effect between PTSD symptoms and romantic relationship satisfaction exists, indicating that improvements in the romantic relationship may lead to the improved well-being of the survivor. Indeed, as romantic partners of PTSD survivors are both negatively impacted by the distress of the survivor, and romantic relationship satisfaction can affect the distress of the PTSD survivor, partners are a key stakeholder for mental health. Unfortunately, theoretical models have not adequately captured the experience of this population to properly illuminate their experience and provide appropriate treatment directives. This paper examines the informal caregiving integrative model to determine its applicability to the romantic partners of PTSD survivors with respect to the determinants, mediators, and outcomes. The current literature on romantic partners is used to evaluate the adequacy of fit, as well as to provide the components unique to partners. Future directions, clinical implications, and limitations of current research are explored based on the results of this review.

## 1. Post-Traumatic Stress Disorder and Romantic Partners

Post-traumatic stress disorder (PTSD) is a debilitating and distressing psychological disorder that can occur following a traumatic event in which an individual is exposed to actual or threatened death, serious injury, or sexual violence [1]. The symptoms of PTSD can create significant distress for survivors, as their lives can change greatly following the trauma (i.e., intrusive thoughts about the event, avoidance of trauma cues, negative mood alterations). As a result of this distress, significant adverse impacts are also experienced by loved ones, e.g., [2]. As with most other mental health conditions, social support is an important protective factor for individuals with PTSD [3]. Because romantic partners are primary social supports for individuals who have experienced trauma, understanding and improving the experience of the partner is essential to understanding and improving the experience of the individual suffering from PTSD.

The romantic partners of individuals with PTSD often incur negative consequences due to the survivor’s PTSD-related distress. Couples in which one partner has PTSD reliably report lower romantic relationship satisfaction than non-PTSD couples [2]. Specifically, these couples are found to have lower intimacy and decreased communication relative to couples without PTSD [4,5]. As such, a bi-directional relationship between relationship functioning and PTSD is observed—relationship functioning may impact PTSD symptoms, while PTSD symptoms may impact the relationship functioning concurrently [6]. The improved functioning of the romantic relationship can lead to the positive improvements in the relationship which can have a noticeable impact on the PTSD-related distress of the survivor. Conversely, negative relationship quality could increase distress.

Certain psychological factors exhibited by the partner can influence the nature of outcomes experienced by the individual with PTSD. For example, beliefs about the trauma survivor’s condition, congruency with their partner’s perceptions of the severity of the PTSD symptoms, and accommodating behaviors (i.e., behaviors that attempt to reduce short-term distress for the PTSD survivor or self [7]) can increase the likelihood of positive or negative outcomes. In fact, partners are so influential that couples therapy for PTSD shows greater improvement in some symptoms relative to individual evidence-based treatment for PTSD, e.g., [8]. Clearly, the partners of PTSD survivors are significant influences on outcomes of both the PTSD survivor and the romantic relationship.

In addition to the survivor and the relationship, it is evident that the partners of PTSD survivors themselves experience distress in their relationship. Partners may experience significant emotional distress, threats of violence, and a burden associated with caring for the PTSD survivor, among other negative outcomes, e.g., [9]. Although undoubtedly important, the distress and outcomes of the partner within PTSD romantic relationship research is currently sparse, with researchers calling for additional attention to this population, e.g., [10].

Unfortunately, much of the research related to partners’ impacts from and influences on PTSD has been atheoretical in nature. This is problematic in that theoretical frameworks for other conditions that impact—and that are impacted by—social support networks may facilitate the understanding of and holistic care for PTSD survivors and their loved ones. To promote clarity in this literature, using frameworks from similar populations may guide future research and inform clinical considerations.

When selecting a framework to elucidate this line of research, one population that shares several characteristics and experiences with partners of PTSD survivors—informal caregivers—may provide insight. Informal caregivers are individuals who care for a family member, friend, or other relative with a chronic condition at home, taking care of several physical and emotional health tasks. Similarly to the loved ones of those with PTSD, informal caregivers assist with the emotional health of their care recipient, perform behaviors and tasks for their care recipient’s betterment, and experience burden due to their role in such relationships [11]. In fact, concepts from informal caregiver research, such as caregiver burden, have been occasionally adapted for the partners of PTSD survivors due to perceived similarity [12]. Yet, there are numerous elements of informal caregivers’ experiences that have not been explicitly examined for trauma survivors and their partners. Due to the similarities these two populations share and the absence of thorough research from a theoretical perspective, it may be helpful to consider the partners of PTSD survivors as akin to caregivers.

Accordingly, the current paper aims to evaluate the applicability of a specific model of informal caregiving to provide a framework for future research and practice regarding the partners of PTSD survivors. This literature will be viewed through the lens of the Informal Caregiving Integrative Model (ICIM) [13] to leverage the findings from caregiver literature and to provide recommendations for future research and clinical practice. Research on the partners of PTSD survivors will be examined representatively through the different aspects of the ICIM to determine the utility of the model as applied to individuals with PTSD and their partners. Throughout the paper, “caregivers” will refer to informal caregivers for individuals with chronic health conditions, “partners” will refer to partners of PTSD survivors, and “survivors” will refer to individuals who have experienced a trauma and have PTSD.

## 2. Informal Caregiving Integrative Model

The ICIM [13] is a theoretical framework aimed at understanding the experience of informal caregivers. This model is used to discuss determinants of caregiver burnout, or exhaustion due to the role of taking care of someone. Burnout has been viewed tri-dimensionally in caregiving research—emotional exhaustion (feeling overloaded, emotionally drained, and unable to continue with the role), depersonalization (i.e., feeling detached or disengaged with the care recipient), and—prophylactically—personal accomplishment (i.e., positive feelings and a sense of fulfillment from caregiving duties). To avoid confusion with the symptom of PTSD referred to as “depersonalization”, we will refer to this dimension as detachment. Important mediators between determinants and burnout in caregivers are coping, relationship satisfaction, and appraisal (i.e., how the caregiver assesses their role). As the experience of caregiver burnout has a significant impact on the caregiver and care recipient [14], it is also important to determine how partners may experience burnout or mitigate their risk of distress. The elements of the ICIM include the determinants of burnout (i.e., caregiver characteristics, caregiving setting, social environment), mediators (i.e., coping, appraisal, relationship quality), specific outcomes (i.e., burnout), and general outcomes (i.e., caregiver and care recipient outcomes)—all will be considered and applied to the PTSD partner literature.

### 2.1. Determinants of Caregiver Burnout

**Figure 1:**
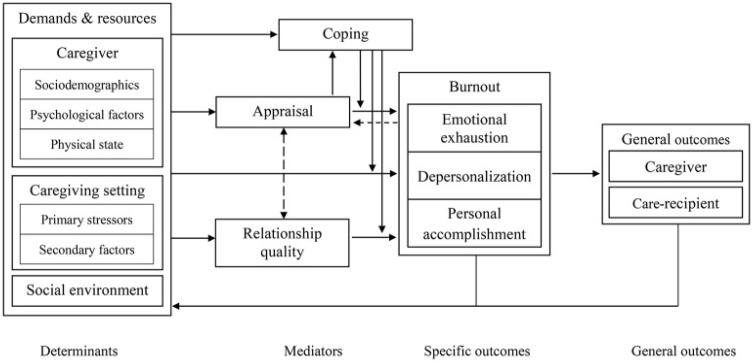
Informal caregiving integrative model. Reprinted with permission from ref [13]. 2019 Gérain, P. (adapted from [13]).

The ICIM posits determinants of what can lead to caregiver burnout, as well as mediators that can lead to positive outcomes. The ICIM (Figure 1) considers different types of factors, defining these as demands (i.e., risk factors for burnout) and resources (i.e., protective factors for burnout). Demands refer to what is asked of the caregiver, while resources refer to what factors or supplies the caregiver has at their disposal to address the demands. Once demands outweigh resources, the caregiver is at greater risk of burnout. In the ICIM, demands and resources reside within three domains—caregiver characteristics, caregiving setting, and social environment.

*Caregiver characteristics—background and sociodemographic factors*. The characteristics of the caregiver provide an understanding of how demands and resources are important determinants of burnout. In caring for chronic health conditions, family caregivers present with background and sociodemographic factors—stable aspects of the caregiver that are innately connected with burnout. Females have an increased likelihood of being a caregiver and experiencing more caregiver tasks [15]. Additionally, caregivers who are older or at a later stage of life tend to experience less burnout [16]. Job status provides some protective effects against burnout [17]. Having fewer identity roles, (e.g., only being a caregiver to a spouse) may be protective relative to individuals with several roles (e.g., being a caregiver to a spouse and a parent to children [18]).

The empirical literature on the partners of PTSD survivors details how background and sociodemographic factors play a role in the care experience for a PTSD survivor, with the literature touching on the factors of biological sex, stage of life, and role status. The female partners of male survivors reliably experience a greater impact on romantic relationship quality when compared to the male partners of female survivors [2]. In a rare study on male partners of female veteran survivors, it was found that the relationship functioning is less impacted than for the female caregivers of male veterans [19]. This may be related to how gender roles in caring are viewed, as caregiving is more often viewed as feminine, and therefore female caregivers may be asked to perform more tasks than male caregivers in equivalent caring situations, as seen in individuals providing care for loved ones with chronic medical conditions [15].

Stage of life is another factor that has an impact on partners. The Partners and spouses of PTSD survivors may be a very young cohort when compared to the spousal caregivers of chronic conditions, as many of the chronic health conditions researched are related to age. Similarly to the broader caregiver literature, younger partners experienced a greater impact on relationship satisfaction and psychological distress [20]. This is likely due to interrupted life plans and beliefs that they should not be providing such extensive care for their partner this early in life.

Role status could also influence the impact on partner caregiving. By way of example, some partners report pressure to buffer children from the survivor in times of distress, guilt in teaching children to avoid trauma-related triggers, and frustration concerning inequity with the survivor in terms of child-rearing [21,22,23]. Conversely, some partners found focusing on childcare at times was a source of respite from their caregiving tasks [22,23]. These findings are similar to chronic health caregiver experiences in some ways, having both positive and negative impacts.

*Caregiver characteristics—psychological factors*. Dispositional, psychological factors may also represent demands and resources that individuals bring to a caregiving context. Caregivers are susceptible to worrying about completing care tasks for the care recipient, fearing the care recipient’s condition worsening, and other concerns of day-to-day life outside of caregiving may increase demands, e.g., [24]. With respect to core personality traits, neuroticism can lead to negative outcomes like depression for caregivers, whereas extraversion may be protective [25]. Unsurprisingly, then, emotional regulation skills are associated with better outcomes for caregivers [26]. In addition to trait-mediated emotionality, cognitions related to self-efficacy are protective against burnout [27]. Low caregiving self-efficacy is associated with risk for burnout [28]. Lastly, intrinsically motivated caregivers tend to have better outcomes compared to caregivers who feel “stuck” in their role [29]. Moreover, heightened rates of psychological disorders have been found among partners of combat veterans, including conditions of insomnia, somatization, and PTSD, among others [30]. Although the presence of psychological disorders is concerning for partners, researchers have yet to fully elucidate the degree to which such distress influences caregiver burnout and relationship satisfaction. Longitudinal research is needed to illuminate the magnitude and directionality of relationships between caregiver psychological distress and relationship quality.

Another factor that has received attention in research regarding partners is the knowledge of the disease. Partners experienced more distress and fear when their partner returned from deployment due to a lack of knowledge of PTSD [31]. Moreover, partners who attributed negative behaviors to PTSD symptoms experience less distress relative to partners who fused PTSD symptoms with the survivor’s personality [32]. Thus, an accurate understanding of PTSD could assist partners with their distress. Knowledge of PTSD can assist partners in attributing why the PTSD survivor may be acting in a certain way, as well as what behaviors may be beneficial. Because these studies focused on combat-related PTSD, it may be useful to see how survivors’ understanding and interpretation of other traumatic events, such as sexual assault and motor vehicle accidents, influence relationship dynamics.

*Caregiver characteristics—physical state*. Finally, with respect to caregiver characteristics that can impact outcomes, the caregiver’s own physical health should be considered. Physical well-being may be a resource for caregivers to utilize in their efforts to navigate the demands of caregiving. Physical illness, including chronic pain, somatic disorders, or their own chronic illness, may impact the ability of the caregiver to perform duties, which reduces their resources to complete other important tasks (i.e., activities of daily living) and may lead to burnout [33]. These illnesses could also impact their mental health negatively, and physical health is of particular concern in older caregivers [34]. These findings indicate that caregivers in good health may have more resources to contribute to caregiving, which may promote better relationship well-being and survivor outcomes.

Regarding research bearing on PTSD caregiver physical health, the partners of veterans with PTSD tend to report more chronic pain and somatic concerns than partners of veterans without PTSD [35,36]. It is possible that the stress associated with caring for another person will lead to physical issues such as these. Although no outcome measures were reported in these studies, in the broader family caregiving literature, caregivers experience more psychological distress while experiencing physical health conditions during caregiving [34]. It is certainly possible that partners will experience similar outcomes. The importance of this finding grows as the cohorts of Vietnam veterans and more recent veterans become older and partners begin to experience more age-related health conditions. Overall, the current research indicates similarity between caregivers and partners in terms of the impacts of physical health on well-being.

*Caregiver setting—primary stressors*. The caregiver setting in the ICIM refers to the context that caregivers find themselves in due to the demands the care recipient requires. This setting is viewed in terms of primary stressors and secondary factors. Primary stressors are associated with objective burden—the workload associated with caregiving [37]. Primary stressors impacting the likelihood of burnout are related to the condition of the care recipient. Duration of caregiving, density of caregiving, and living with the care recipient are all either inconclusive or are found to have little impact on burnout [38]. Primary stressors most associated with caregiver burnout are related to the health condition of the care recipient. Functional impairment due to disease has been found to result in increased subjective burden and burnout [38]. Comorbid conditions, such as mental health conditions in accordance with physical health conditions, have increased burnout likelihood for parental caregivers [39]. Lastly, the emotional well-being of the care recipient is associated with caregiver burnout as well [40]. Overall, primary stressors of functional impairment, disease severity, comorbid conditions, and the emotional well-being of the care recipient contribute the most to burnout.

With respect to individuals with PTSD, one study identified that the functional impairment of veteran survivors contribute to emotional distress and caregiver burden experienced by the partner [41]. However, burden was the mediating factor between veteran impairment and emotional distress experienced by the partner, implying that burden causes emotional distress. This finding is unclear though, as the Caregiver Burden Index CBI [42] used in the study assesses both objective burden and emotional distress. As such, this mediating relationship may be confounded by construct overlap of the CBI and emotional distress rather than burden leading to emotional distress. Future research may also benefit from the different definitions of functional impairment for PTSD survivors. The severity of disease has been found to significantly impact the partner of the PTSD survivor, such that more severe PTSD presentations lead to increased burden [12], psychological distress, and relationship quality [2,12].

Trauma type could be a unique primary stressor for this population. As discussed, much of the literature studies combat veterans with PTSD and the impact on their partners. When compared to civilian trauma, veteran dyads experience more relationship distress and aggression, which likely contribute to the relationship dissatisfaction [43]. Conceivably, different types of trauma precipitating PTSD may have differential impacts on relationship functioning. For example, the partners of sexual assault survivors may have novel difficulties due to the impacts on physical intimacy experienced by the survivors, which could impact intimacy with a romantic partner. The symptom cluster of re-experiencing has often had minimal impact on romantic relationship functioning in veterans due to its clear link to the traumatic event [32]. It is possible, however, that this would be different for sexual assault survivors. Intrusions during sexual activity may impact sexual functioning, and therefore romantic satisfaction for some partners. In addition to physical intimacy concerns, sexual assault survivors may have enhanced difficulty in trusting relationship partners due to past betrayal in romantic contexts. More research is necessary to determine how PTSD manifests in different relationships due to different trauma types.

*Caregiver setting—secondary factors*. Secondary factors describe the impact of functional impairment of the primary stressors that is related to caregiver burnout. Some of these stressors include less free time for the caregiver, reduced social life, and reducing valued activities due to care recipient’s condition [44]. These can all result in increased distress and reduction in respite from their caregiver role. Secondary factors can lead to some resentment of the care recipient, which may impact the relationship [39]. Preliminary evidence suggests that the presence of primary stressors may create social support opportunities by creating a new identity to share, such as in caregiver support groups [45]. This provides the lone resource in secondary factors.

Partners of individuals with PTSD may experience many similarities with caregivers for other conditions regarding secondary factors. Social isolation may result from feelings of guilt leaving the PTSD survivor alone [46]. Partners also tend to experience fear-related to perceptions of physical threat [47]. Specifically, one study found that 60% of PTSD partners viewed their partner as a physical threat to themselves [9]. Importantly, these findings are primarily from military couples, which may not be comparable to the physical threats perceived by civilian PTSD partner populations [43]. Thus, it is important to understand how physical threats and safety is experienced differently for these partners. Indeed, what we know about the experiences of partners currently map on well to the ICIM regarding functional impact, social isolation, and resentment, while the additional aspect of threats to physical safety could be unique to PTSD-related caregiving.

Prophylactically, partners often experience some resources of social support due to identification as a partner of a PTSD survivor. Support groups have been developed for families of veterans with PTSD, which intend to increase social support for families, partners, and survivors, e.g., [48]. Specifically, survivors experience reductions in PTSD and improvements in relationship satisfaction, while partners (and other family members) experience improvements in social support and relationship satisfaction after being involved in formal support programs [49]. The support groups are often run by Veteran’s Affairs services (VA), which provides an additional resource to partners of veterans, e.g., [48,49]. This access allows for support from others to be built by group members who understand their situation. Unfortunately, this exists as a resource that partners of civilian PTSD survivors cannot access, as these are created for veteran families. Nonetheless, partners who attend support groups do experience a similar positive impact that caregivers do based on the access to support groups.

*Social environment*. The social environment for caregivers refers to several sociocultural aspects of relational demands or resources that the caregiver may be privy to. The cultural background of the caregiver may set expectations about how the individual views the caregiving role—as duty or as unexpected [50]. The cultural perspectives of caregiving can assist caregivers, as relatives may assist in caregiving duties in collectivist cultures [51]. However, collectivist culture can increase belief of being forced into the role due to cultural expectation [50]. Clearly, culture affects the caregiver’s appraisal of their role. Social support for caregivers can provide important resources to caregivers—whether that be formal support services to assist with activities of daily living and respite [52] or provide spaces to have emotional support from family and friends [53].

Culture may dictate expectations on what partners are expected to do when their spouse is in need of care. One study that did intend to show how culture influences PTSD partners found that culture in Iran may compel the female spouse to take care of the veteran husband who fought in a holy war as a duty to show devotion to the community [54]. As these partners experienced significant psychological distress, it is recommended to explore whether a sense of duty to the partner versus the community provides differential results, as a sense of duty to combat veterans can be protective in the United States [55]. Although sparse, cultural influence in one study was determined to be comparable to an outcome of caregiver literature, e.g., [50]. Future studies could examine whether partners can experience protective factors as well.

Social support could be an important aspect of the experience of partners with PTSD; however, research has been sparse. Social isolation and the importance of social support have been highlighted as needs and concerns qualitatively, e.g., [46,56]. Still, more needs to be accomplished in this area of the literature. Focus on social support for survivors has received deserved attention, but the investigation of the impacts of social support of PTSD partners, who are experiencing isolation, psychological distress, and relationship dysfunction, would be useful. Improved social support may well provide improved motivation and decreases in burnout likelihood.

### 2.2. Mediators of Caregiver Burnout

*Coping*. The ICIM suggests that factors such as an individual’s ways of coping—their approach to the stressful caregiving situation they are placed in—can provide a mediating effect between determinants and burnout. Coping in the broader caregiver literature is typically focused on emotional regulation as a disposition rather than responses to situations, which is considered a gap in the literature [13]. Thus, it is possible that partner literature may provide some insight into caregiver literature for this element of the ICIM.

The strongest example of coping found for the partners of individuals with PTSD is accommodating behaviors. Due to the distress experienced from PTSD symptoms, partners may participate in behaviors that reduce distress in the moment. Partners tend to perform accommodation behaviors primarily with the intention of preserving the relationship or avoiding conflict, with a small portion of behaviors performed for an ill-informed rationale that the avoidance of PTSD symptoms and trauma reminders will improve the condition of the partner in the long term [7]. Although well intentioned, these behaviors often result in short-term relief but negative long-term impacts on the partner’s distress, the survivor’s PTSD symptoms, and the romantic relationship functioning [57,58]. Additionally, partners providing accommodating behaviors to survivors with a lower severity of PTSD experience has a larger impact on their emotional intimacy when collecting daily diary information [59]. This effect has been found to have an increasing impact over time, with partners who accommodate to avoid conflict having the worst outcomes in terms of depression [60].

Indeed, accommodating partners are significant stakeholders in the outcomes of themselves, the survivor, and the relationship. The experience of “walking on eggshells” for partners is well documented [22]. An important question focuses on the accuracy of the partners’ perceptions of the utility in their accommodation behaviors—that is, are partners correct that their accommodating behaviors are avoiding conflict and reducing distress for the survivor in the moment? Data would suggest that some partners accommodate more than necessary, leading to worse outcomes [59]. It is important to consider the experience of the survivor. Understandably, PTSD symptoms that are accommodated, and therefore avoided, will likely persist, as treatment modalities suggest, e.g., [61]. Even so, this behavior is linked to reductions in the survivor’s relationship satisfaction as well, so the mechanism that reduces the relationship satisfaction for the survivor is problematic. An alternative explanation comes from broader caregiver literature. Palliative care patients experience self-perceived burden on their spouses and family due to the physical restrictions they experience and the emotional distress they cause their spouses due to their condition [62]. Applied to the partners of PTSD survivors, the presence of accommodating behaviors may indicate that the survivor is a burden on their partner. Secrecy or avoiding discussing the event has been suggested as a possible unique accommodation for PTSD survivors, especially in cases of sexual assault [63]. Overall, accommodating behaviors to avoid negative emotional outcomes, although intuitive and well intentioned, tend to prolong or exacerbate PTSD symptoms and adversely impact relationships.

*Caregiver appraisal*. The appraisal of their role is an important mediator for caregivers regarding burnout. Specifically, this refers to the caregiver’s evaluation of the demands of their role given their resources. Simply put, caregivers perceiving their role to be excessively demanding have a greater likelihood of burnout, e.g., [28]. In fact, subjective burden is believed to be the most critical caregiver attribute in terms of experiencing caregiver burnout [64]. Caregiver appraisal can include feeling trapped in the role of caregiving, which leads to poorer outcomes [65]. Alternatively, a positive view of the caregiver role can lead to a decreased likelihood of burnout, e.g., [66]. This is to be expected—an individual who finds a demanding role more intrinsically valuable will inherently perceive the role as less burdensome, even if the demands are objectively equal. With respect to applicability of this mediator to partners of individuals with PTSD, as mentioned earlier, partners of veterans who view the survivor’s service as meaningful experienced better relationship outcomes [55]. Although less well extensively studied among partners of individuals with PTSD relative to other types of caregivers, it is likely that the appraisal of the value of one’s role as caregiver relative to perceived resources and demands would influence burnout in the same manner that has been shown in the broader caregiving literature. Thus, there is a significant need to focus on how partners appraise their role as a caregiver for their partner to facilitate their understanding of the applicability of ICIM tenets to PTSD caregiving.

*Relationship quality*. The quality of the relationship between the caregiver and care recipient is an important aspect of caregiving that is important when considering burnout. The poor relationship quality increases the risk of burnout. An important factor that can lead to experiencing a poor relationship quality are feelings of inequity in the relationship in spousal caregiving in particular [67]. Meanwhile, a positive relationship quality can be a protective factor, and importantly, caregiver appraisal may be connected to their romantic relationship quality. This can be partially explained by the aspects of relationship quality, such as commitment and communication [67].

Relationship quality in the partners of those with PTSD has been a key focus of research in this population. As previously discussed, partners have been found to experience decreased relationship quality in both civilian and military populations, e.g., [2] as well as greater impacts on relationship quality as the PTSD severity increases [68], which are associated with factors such as caregiver burden [56] and the appraisal of the survivor’s PTSD symptoms [69]. Therefore, the partner’s relationship quality disruptions conform to similar disruptions identified in the caregiver literature and the ICIM.

More specifically, partners, much like caregivers, may experience disruptions in important relationship factors, such as communication and intimacy. Partners may restrict sharing their needs or concerns out of fear of distressing the survivor [70]. Communication disruptions are often credited to emotional numbing symptoms, which many approaches in couples therapy target for PTSD survivors [71]. Longitudinal evidence for PTSD due to a severe motor vehicle accident suggest that increased emotional numbing symptoms may be predicted by dysfunctional communication, which in turn may be associated with avoidance symptoms and PTSD severity overall [72]. Thus, it is possible that communication disruptions can worsen emotional numbing, which heavily contributes to decreases in relationship functioning and satisfaction overall [73]. In couples therapy approaches, communication enhancement is integral to the therapy sessions, which facilitates an understanding of the survivor’s experiences [74]. In terms of intimacy, partners reported that greater emotional intimacy from the survivor was a key desired change in their relationship [75]. Paradoxically, partners fear intimacy and being close with the survivor [76]. This discrepancy of desiring more intimacy from the PTSD survivor while simultaneously fearing intimacy may create distress for the survivor. Sexual functioning may decrease for both veterans [77] and sexual assault survivors [78], indicating the disruption of physical intimacy for partners. Overall, communication and intimacy are at risk of disruption for partners, which may be aspects that are important for relationship functioning and coincide with similar experiences from caregivers.

A unique form of communication for partners is the construct of disclosure—providing details about a traumatic event to someone else. The effects can vary in function of the perception of the partner’s reaction—positive reactions from partners can lead to further trauma disclosure in sexual assault survivors [79]. This, as discussed above, can be critical for improving the communication and understanding of the PTSD survivor for the partner. Positive, supportive responses were associated with increased sexual satisfaction among child sexual abuse survivors, while negative responses resulted in decreased romantic relationship satisfaction for both partners [80]. Without sharing the nature of PTSD symptoms and traumatic experiences that gave rise to them, partners may incorrectly conclude that the symptoms are a result of the personality rather than the disease. Research with veteran populations indicates that incorrectly attaching PTSD symptoms to the survivor’s personality is harmful to relationship quality [32]. In contrast to the experience of partners of sexual assault survivors, the disclosure of details related to combat-related trauma for PTSD survivors has a negative effect on the partner’s psychological distress, but not on the relationship’s distress [81]. It is possible that it may be less important for combat veterans to disclose trauma because their partner likely is aware that traumatic events occurred while they were deployed, and the result of these events explain the symptoms for the survivor. Conversely, civilian PTSD survivors may need to provide context for their emotional distress for their partners. More research on the psychological distress of partner disclosure with different types of trauma may be illuminating to determine why this occurs in veteran populations. Overall, trauma-related disclosure is likely an important influence on relationship quality that has implications for the applicability of the ICIM for this population.

### 2.3. Caregiver Burnout

The determinants and mediators of caregiver burnout proposed by the Informal Caregiving Integrative Model that have been discussed thus far can influence three different dimensions of burnout—emotional exhaustion, detachment, and personal accomplishment. The negative dimensions—emotional exhaustion and detachment—must outweigh the positive dimension of personal accomplishment for burnout to occur (although positive feelings in caregiving may co-occur and are generally independent of negative feelings [82]). Personal accomplishment may refer to benefit for an individual or for a deepening relationship with the care recipient.

Though well established in many caregiving populations, burnout is a construct that has not been as extensively examined in partners of individuals with PTSD. Only two studies have attempted to measure caregiver burnout in partners of PTSD survivors [83,84]. Based on those studies, it appears that partners experience greater burnout when the subjective burden is high, and worse outcomes result from both partners suffering from PTSD [83]. Additionally, partners experienced improvements in caregiver burnout following a family-based intervention [84]. Unfortunately, these studies focused on and measured the negative facets of caregiver burnout (i.e., experiences akin to exhaustion and attachment) but failed to assess the positive outcomes proposed by the ICIM—i.e., personal achievement related to caregiving, which can provide buffering effects to burnout [82]. Generally, the PTSD caregiving literature remains underdeveloped with respect to explicitly examining burnout per se as opposed to related constructs such as relationship satisfaction and emotional distress. Due to the paramount significance of burnout in the broader caregiver literature, this is a clear future direction for studies involving the partners of PTSD survivors.

The partners of PTSD survivors do experience some positive aspects of their role even if studies have not explicitly examined personal accomplishment as a caregiver. Positive experiences do not necessarily constitute personal achievement, as difficult or stressful situations could also lead to that conclusion—however, it is useful to discuss that positive affect and emotions do exist for partners about their role. For instance, some spouses have indicated improved connection due to caring for the PTSD survivor because they began showing vulnerability, or have viewed their partners more favorably due to the strength they show during their struggle [85]. Generally, however, the relative dearth of literature on personal accomplishment may miss a great portion of what some partners experience in their relationships. It may well be a reason for many partners staying in a difficult relationship. More research focused on personal accomplishment as opposed to a positive affect broadly will assist in understanding whether personal accomplishment as a caregiver exerts a buffering effect for the partners of PTSD survivors.

### 2.4. General Outcomes

The extent to which one experiences caregiver burnout can lead to numerous outcomes for both the caregiver and the care recipient. Experiencing significant burnout is associated with elevated distress and negative emotions, such as anxiety, depression, psychosomatic consequences, and poor physical health [86]. For care recipients, unfortunately, there are associations with burnout and abuse, which can include neglect, physical violence, and verbal violence [87]. Thus, for both the well-being of the caregiver and care recipient, it is clear that burnout is a crucial element in the model of caregiving.

*Caregiver outcomes*. Partners of PTSD survivors are at risk of negative outcomes tied to physical and psychological health. Although not a frequently assessed variable, there have been documented associations of PTSD symptom severity with somatic symptoms [36] and chronic pain [35] for partners. Additionally, partners of veterans with PTSD may be at risk to cardiovascular disease due to the increased cardiovascular reactivity of PTSD survivors during conflict [88]. Psychological associations for partners have been discussed for partners significantly throughout this review, such as general psychological distress [9], depression and anxiety, e.g., [35], sleep problems [36], and many other mental health conditions [30].

In addition to some of these intrapersonal negative outcomes, intimate partner violence is of concern for this population. Many partners have described violence and fears of physical aggression from veteran partners with PTSD [89]. A significant percentage of partners have been found to experience threats to their physical safety [9]. Symptom severity and symptom clusters of hyperarousal are associated with intimate partner violence [4]. It is unclear whether and to what degree partner violence is heightened in other, non-combat trauma populations, or whether these issues are unique to military related PTSD.

*Care recipient outcomes*. A viable outcome for PTSD survivors is improvement in PTSD symptoms, which is often not a possibility for many chronic health conditions that inform the caregiving literature, where the disease is progressive and incurable. It has been suggested that trauma survivors with higher perceived support from their partners have a reduced likelihood of developing PTSD [90]. Additionally, romantic relationship quality has been suggested to have a bi-directional effect with PTSD, such that, while PTSD symptoms can decrease relationship quality, the relationship quality can also improve the PTSD symptoms, and specifically symptom clusters, over time [6]. Concretely, improvements in relationship quality may impact factors such as communication and intimacy that are inherently related to PTSD symptoms. In addition to impacting symptoms in daily life, partners’ participation in PTSD treatment may assist in positive outcomes for the survivor. Cognitive behavioral couples therapy for PTSD has been found to better address emotional numbing clusters for survivors relative to individual PTSD treatments [8]. Thus, partner involvement in the treatment of PTSD may produce treatment gains above and beyond those which occur in the context of individual evidence-based treatment for PTSD. More research is needed to connect these outcomes to burnout. Ideally, positive outcomes—at both the individual and partner levels—can be enhanced by considering and focusing on caregiver dynamics during treatment. Clearly, broad social support—whether from partners, therapists, or groups—can greatly facilitate healing and growth following trauma and difficult circumstances.

## 3. Future Research Directions

Though the ICIM appears to have important implications for understanding caregiving impacts within trauma populations, research samples have been heavily skewed to couples with combat-related PTSD. Although there are a few notable exceptions, the partners of civilians with PTSD have been largely ignored. As such, the generalizability of the ICIM to non-combat trauma populations remains unclear and important differences may exist as a function of trauma type. For example, the partners of sexual assault survivors may have specific relationship functioning impairments due to the nature of the trauma in terms of sexual functioning and communication. Additionally, many sociodemographic and background factors could provide important future research directives in this population. Examining some variables that have received little attention, such as physical health, personality traits, cognitions of self-efficacy, and culture should provide a richer understanding of determining which partners are at a greater risk of negative outcomes. One sociodemographic factor that has not received much attention for caregivers or partners is sexual orientation. All studies reported throughout this review have been based on heterosexual couples. Therefore, some results may not be generalizable to non-heterosexual couples due to the heteronormativity of current literature. Lastly, two important elements of the ICIM have been generally neglected or underdeveloped in the literature about partners of PTSD survivors—appraisal and caregiver burnout. Although the appraisal of the PTSD survivor has occurred, this literature has ignored how the partner appraises their role—how valuable do they find their experience in assisting their partner with PTSD? Similarly, caregiver burden has been examined in this literature, but rarely has burnout been effectively examined as a tridimensional construct—specifically, personal achievement derived from being a caregiver has been largely ignored. Understanding the positive and motivating aspects of caring for a partner with PTSD will surely inform PTSD caregiving outcomes as well as how best to support partners of those who have been impacted by trauma.

## 4. Clinical Implications

Research findings to date have clinical implications for partners. First, using the ICIM, the most promising targets for change are the mediators—relationship quality, coping, and the appraisal of the caregiving role. To improve the relationship quality, it is recommended that partners are involved in couples therapy with the PTSD survivor—these treatments show positive impacts on relationship quality [71]. Focusing efforts on appraisal and the coping of the partner may be fruitful in improving the experience for the partners of helping survivors, which could be pursued in both couples and individual therapy. Additionally, treating negative psychological outcomes would allow the partner to have more resources to utilize in their caregiving role. With respect to targeting the appraisal of one’s caregiving role, cognitive restructuring may be particularly useful to facilitate reframing the situation that the partner finds themselves in. Teaching partners what coping efforts are effective and what coping efforts are ineffective, such as accommodation, could also be critical to success.

## 5. Conclusions

Based on the reviewed literature, the experience of caregivers for broader health conditions—through determinants, mediators, and outcomes identified by the ICIM—holds great applicability for understanding and improving the plight of those caring for individuals with PTSD. This perspective shifts the focus from caregivers as a peripheral consideration to the necessity of explicitly considering and incorporating supportive others in a comprehensive care framework. Doing so stands to improve the outcomes for the individual with PTSD, their loved ones, and their collective wellbeing. Holistic care for individuals with PTSD requires greater attention to their relationships and supports. Caregivers can experience tremendous strain and burnout, but they are also well positioned to enhance trauma survivors’ functioning and well-being. Greater understanding of the experience of partners, including elements that are relevant to preventing negative outcomes, can be achieved by considering the implications of the ICIM which have proven to be beneficial for other caregiving populations. 

## References

[1] American Psychiatric Association (2013). Diagnostic and Statistical Manual of Mental Disorders.

[2] Lambert J.E., Engh R., Hasbun A., Holzer J. (2012). Impact of posttraumatic stress disorder on the relationship quality and psychological distress of intimate partners: A meta-analytic review. J. Fam. Psychol..

[3] Wang Y., Chung M.C., Wang N., Yu X., Kenardy J. (2021). Social support and posttraumatic stress disorder: A meta-analysis of longitudinal studies. Clin. Psychol. Rev..

[4] Birkley E.L., Eckhardt C.I., Dykstra R.E. (2016). Posttraumatic stress disorder symptoms, intimate partner violence, and relationship functioning: A meta-analytic review. J. Trauma. Stress.

[5] DiMauro J., Renshaw K.D. (2019). PTSD and relationship satisfaction in female survivors of sexual assault. Psychol. Trauma Theory Res. Pract. Policy.

[6] LeBlanc N.J., Dixon L., Robinaugh D.J., Valentine S.E., Bosley H.G., Gerber M.W., Marques L. (2016). PTSD and romantic relationship satisfaction: Cluster-and symptom-level analyses. J. Trauma. Stress.

[7] Renshaw K.D., Allen E.S., Fredman S.J., Giff S.T., Kern C. (2020). Partners’ motivations for accommodating posttraumatic stress disorder symptoms in service members: The reasons for accommodation of PTSD scale. J. Anxiety Disord..

[8] Macdonald A., Pukay-Martin N.D., Wagner A.C., Fredman S.J., Monson C.M. (2016). Cognitive–behavioral conjoint therapy for PTSD improves various PTSD symptoms and trauma-related cognitions: Results from a randomized controlled trial. J. Fam. Psychol..

[9] Manguno-Mire G., Sautter F., Lyons J., Myers L., Perry D., Sherman M., Glynn S., Sullivan G. (2007). Psychological distress and burden among female partners of combat veterans with PTSD. J. Nerv. Ment. Dis..

[10] Campbell S.B., Renshaw K.D. (2018). Posttraumatic stress disorder and relationship functioning: A comprehensive review and organizational framework. Clin. Psychol. Rev..

[11] Carretero S., Garcés J., Ródenas F., Sanjosé V. (2009). The informal caregiver’s burden of dependent people: Theory and empirical review. Arch. Gerontol. Geriatr..

[12] Calhoun P.S., Beckham J.C., Bosworth H.B. (2002). Caregiver burden and psychological distress in partners of veterans with chronic posttraumatic stress disorder. J. Trauma. Stress.

[13] Gérain P., Zech E. (2019). Informal caregiver burnout? Development of a theoretical framework to understand the impact of caregiving. Front. Psychol..

[14] Brodaty H., Donkin M. (2009). Family caregivers of people with dementia. Dialogues Clin. Neurosci..

[15] Xiong C., Biscardi M., Astell A., Nalder E., Cameron J.I., Mihailidis A., Colantonio A. (2020). Sex and gender differences in caregiving burden experienced by family caregivers of persons with dementia: A systematic review. PLoS ONE.

[16] Carter J.H., Lyons K.S., Stewart B.J., Archbold P.G., Scobee R. (2010). Does age make a difference in caregiver strain? Comparison of young versus older caregivers in early-stage Parkinson’s disease. Mov. Disord..

[17] Kokurcan A., Özpolat A.G.Y., Göğüş A.K. (2015). Burnout in caregivers of patients with schizophrenia. Turk. J. Med. Sci..

[18] Lindt N., van Berkel J., Mulder B.C. (2020). Determinants of overburdening among informal carers: A systematic review. BMC Geriatr..

[19] Knopp K., Wrape E.R., McInnis R., Khalifian C.E., Rashkovsky K., Glynn S.M., Morland L.A. (2022). Posttraumatic stress disorder and relationship functioning: Examining gender differences in treatment-seeking veteran couples. J. Trauma. Stress.

[20] Renshaw K.D., Rodebaugh T.L., Rodrigues C.S. (2010). Psychological and marital distress in spouses of Vietnam veterans: Importance of spouses’ perceptions. J. Anxiety Disord..

[21] Chapin M. (2011). Family resilience and the fortunes of war. Soc. Work Health Care.

[22] Beks T. (2016). Walking on Eggshells: The Lived Experience of Partners of Veterans with PTSD. Qual. Rep..

[23] Wick S., Nelson Goff B.S. (2014). A qualitative analysis of military couples with high and low trauma symptoms and relationship distress levels. J. Couple Relatsh. Ther..

[24] Kaddour L., Kishita N. (2020). Anxiety in informal dementia carers: A meta-analysis of prevalence. J. Geriatr. Psychiatry Neurol..

[25] Kim S.K., Park M., Lee Y., Choi S.H., Moon S.Y., Seo S.W., Park K.W., Ku B.D., Han H.J., Park K.H. (2017). Influence of personality on depression, burden, and health-related quality of life in family caregivers of persons with dementia. Int. Psychogeriatr..

[26] Murfield J., Moyle W., O’Donovan A., Ware R.S. (2020). The role of self-compassion, dispositional mindfulness, and emotion regulation in the psychological health of family carers of older adults. Clin. Gerontol..

[27] Au A., Lai M.K., Lau K.M., Pan P.C., Lam L., Thompson L., Gallagher-Thompson D. (2009). Social support and well-being in dementia family caregivers: The mediating role of self-efficacy. Aging Ment. Health.

[28] Cuijpers P., Stam H. (2000). Burnout among relatives of psychiatric patients attending psychoeducational support groups. Psychiatr. Serv..

[29] Dombestein H., Norheim A., Lunde Husebø A.M. (2020). Understanding informal caregivers’ motivation from the perspective of self-determination theory: An integrative review. Scand. J. Caring Sci..

[30] Steenkamp M.M., Corry N.H., Qian M., Li M., McMaster H.S., Fairbank J.A., Marmar C.R. (2018). Prevalence of psychiatric morbidity in United States military spouses: The millennium cohort family study. Depress. Anxiety.

[31] McLean H.B. (2006). A Narrative Study of the Spouses of Traumatized Canadian Soldiers. Doctoral Dissertation.

[32] Renshaw K.D., Allen E.S., Carter S.P., Markman H.J., Stanley S.M. (2014). Partners’ attributions for service members’ symptoms of combat-related posttraumatic stress disorder. Behav. Ther..

[33] Truzzi A., Valente L., Ulstein I., Engelhardt E., Laks J., Engedal K. (2012). Burnout in familial caregivers of patients with dementia. Braz. J. Psychiatry.

[34] Blyth F.M., Cumming R.G., Brnabic A.J., Cousins M.J. (2008). Caregiving in the presence of chronic pain. J. Gerontol. Ser. A Biol. Sci. Med. Sci..

[35] Koic E., Muzinic-Masle L., Franciskovic T., Vondracek S., Djordjevic V., Per-Koznjak J., Prpic J. (2002). Chronic pain in the wives of the Croatian war veterans treated for PTSD. Eur. Psychiatry.

[36] Dirkzwager A.J., Bramsen I., Adèr H., van der Ploeg H.M. (2005). Secondary traumatization in partners and parents of Dutch peacekeeping soldiers. J. Fam. Psychol..

[37] Montgomery R.J.V., Gonyea J.G., Hooyman N.R. (1985). Caregiving and the experience of subjective and objective burden. Fam. Relat..

[38] Chiao C.Y., Wu H.S., Hsiao C.Y. (2015). Caregiver burden for informal caregivers of patients with dementia: A systematic review. Int. Nurs. Rev..

[39] Gérain P., Zech E. (2018). Does informal caregiving lead to parental burnout? Comparing parents having (or not) children with mental and physical issues. Front. Psychol..

[40] Egbert N., Dellmann-Jenkins M., Smith G.C., Coeling H., Johnson R.J. (2008). The emotional needs of care recipients and the psychological well-being of informal caregivers: Implications for home care clinicians. Home Healthc. Now.

[41] Dekel R., Solomon Z., Bleich A. (2005). Emotional distress and marital adjustment of caregivers: Contribution of level of impairment and appraised burden. Anxiety Stress Coping.

[42] Caserta M.S., Lund D.A., Wright S.D. (1996). Exploring the Caregiver Burden Inventory (CBI): Further evidence for a multidimensional view of burden. Int. J. Aging Hum. Dev..

[43] Taft C.T., Watkins L.E., Stafford J., Street A.E., Monson C.M. (2011). Posttraumatic stress disorder and intimate relationship problems: A meta-analysis. J. Consult. Clin. Psychol..

[44] Otis-Green S., Juarez G. (2012). Enhancing the social well-being of family caregivers. Semin. Oncol. Nurs..

[45] Sakakibara K., Kabayama M., Ito M. (2015). Experiences of “endless” caregiving of impaired elderly at home by family caregivers: A qualitative study. BMC Res. Notes.

[46] Sherman M.D., Zanotti D.K., Jones D.E. (2005). Key elements in couples therapy with veterans with combat-related posttraumatic stress disorder. Prof. Psychol. Res. Pract..

[47] Solomon Z., Dekel R., Zerach G. (2008). The relationships between posttraumatic stress symptom clusters and marital intimacy among war veterans. J. Fam. Psychol..

[48] Sherman M.D., Perlick D.A., Straits-Tröster K. (2012). Adapting the multifamily group model for treating veterans with posttraumatic stress disorder. Psychol. Serv..

[49] Fischer E.P., Sherman M.D., Han X., Owen Jr R.R. (2013). Outcomes of participation in the REACH multifamily group program for veterans with PTSD and their families. Prof. Psychol. Res. Pract..

[50] Pharr J.R., Dodge Francis C., Terry C., Clark M.C. (2014). Culture, caregiving, and health: Exploring the influence of culture on family caregiver experiences. Int. Sch. Res. Not..

[51] Andruske C.L., O’Connor D. (2020). Family care across diverse cultures: Re-envisioning using a transnational lens. J. Aging Stud..

[52] Schoenmakers B., Buntinx F., DeLepeleire J. (2010). Supporting the dementia family caregiver: The effect of home care intervention on general well-being. Aging Ment. Health.

[53] Anjos K.F.D., Boery R.N.S.D.O., Pereira R., Pedreira L.C., Vilela A.B.A., Santos V.C., Rosa D.D.O.S. (2015). Association between social support and quality of life of relative caregivers of elderly dependents. Cienc. Saude Coletiva.

[54] Ahmadi K., Azampoor-Afshar S., Karami G., Mokhtari A. (2011). The association of veterans’ PTSD with secondary trauma stress among veterans’ spouses. J. Aggress. Maltreatment Trauma.

[55] Bergmann J.S., Renshaw K.D., Allen E.S., Markman H.J., Stanley S.M. (2014). Meaningfulness of service and marital satisfaction in Army couples. J. Fam. Psychol..

[56] Skowronski S. (2019). PTSD on Relational and Sexual Functioning and Satisfaction: Evaluating the Effects of PTSD, Emotional Numbing, Moral Injury, and Caregiver Burden. Ph.D. Dissertation.

[57] Fredman S.J., Vorstenbosch V., Wagner A.C., Macdonald A., Monson C.M. (2014). Partner accommodation in posttraumatic stress disorder: Initial testing of the Significant Others’ Responses to Trauma Scale (SORTS). J. Anxiety Disord..

[58] Kenny J.J., Allen E., Renshaw K., Bhalla A., Fredman S.J. (2021). Two perspectives on accommodation of PTSD symptoms: Partners versus service members. Couple Fam. Psychol. Res. Pract..

[59] Campbell S.B., Renshaw K.D. (2019). Daily posttraumatic stress disorder symptom accommodation and relationship functioning in military couples. Fam. Process.

[60] Fredman S.J., Le Y., Renshaw K.D., Allen E.S. (2022). Longitudinal Associations among Service Members’ PTSD Symptoms, Partner Accommodation, and Partner Distress. Behav. Ther..

[61] Resick P.A., Monson C.M., Chard K.M. (2016). Cognitive Processing Therapy for PTSD: A Comprehensive Manual.

[62] Gudat H., Ohnsorge K., Streeck N., Rehmann-Sutter C. (2019). How palliative care patients’ feelings of being a burden to others can motivate a wish to die. Moral challenges in clinics and families. Bioethics.

[63] Reuman L., Thompson-Hollands J. (2020). Family accommodation in PTSD: Proposed considerations and distinctions from the established transdiagnostic literature. Clin. Psychol. Sci. Pract..

[64] Takai M., Takahashi M., Iwamitsu Y., Oishi S., Miyaoka H. (2011). Subjective experiences of family caregivers of patients with dementia as predictive factors of quality of life. Psychogeriatrics.

[65] Nemati S., Rassouli M., Ilkhani M., Baghestani A.R. (2018). Perceptions of family caregivers of cancer patients about the challenges of caregiving: A qualitative study. Scand. J. Caring Sci..

[66] Kajiwara K., Nakatani H., Ono M., Miyakoshi Y. (2015). Positive appraisal of in-home family caregivers of dementia patients as an influence on the continuation of caregiving. Psychogeriatrics.

[67] Braun M., Scholz U., Bailey B., Perren S., Hornung R., Martin M. (2019). Dementia caregiving in spousal relationships: A dyadic perspective. Aging Ment. Health.

[68] Riggs D.S., Byrne C.A., Weathers F.W., Litz B.T. (1998). The quality of the intimate relationships of male Vietnam veterans: Problems associated with posttraumatic stress disorder. J. Trauma. Stress.

[69] Renshaw K.D., Caska C.M. (2012). Relationship distress in partners of combat veterans: The role of partners’ perceptions of posttraumatic stress symptoms. Behav. Ther..

[70] Grimesey J. (2009). The Impact of Combat Trauma on Veterans’ Family Members: A Qualitative Study. Doctoral Dissertation.

[71] Monson C.M., Fredman S.J., Macdonald A., Pukay-Martin N.D., Resick P.A., Schnurr P.P. (2012). Effect of cognitive-behavioral couple therapy for PTSD: A randomized controlled trial. JAMA.

[72] Fredman S.J., Beck J.G., Shnaider P., Le Y., Pukay-Martin N.D., Pentel K.Z., Monson C.M., Simon N.M., Marques L. (2017). Longitudinal associations between PTSD symptoms and dyadic conflict communication following a severe motor vehicle accident. Behav. Ther..

[73] Campbell S.B., Renshaw K.D. (2013). PTSD symptoms, disclosure, and relationship distress: Explorations of mediation and associations over time. J. Anxiety Disord..

[74] Pukay-Martin N.D., Fredman S.J., Martin C.E., Le Y., Haney A., Sullivan C., Monson C.M., Chard K.M. (2022). Effectiveness of cognitive behavioral conjoint therapy for posttraumatic stress disorder (PTSD) in a US Veterans Affairs PTSD clinic. J. Trauma. Stress.

[75] LaMotte A.D., Taft C.T., Reardon A.F., Miller M.W. (2015). Veterans’ PTSD symptoms and their partners’ desired changes in key relationship domains. Psychol. Trauma Theory Res. Pract. Policy.

[76] Riggs D.S. (2014). Traumatized relationships: Symptoms of posttraumatic stress disorder, fear of intimacy, and marital adjustment in dual trauma couples. Psychol. Trauma Theory Res. Pract. Policy.

[77] Rosebrock L., Carroll R. (2017). Sexual function in female veterans: A review. J. Sex Marital Ther..

[78] Bigras N., Godbout N., Briere J. (2015). Child sexual abuse, sexual anxiety, and sexual satisfaction: The role of self-capacities. J. Child Sex. Abus..

[79] DiMauro J., Renshaw K.D. (2021). Trauma-related disclosure in sexual assault survivors’ intimate relationships: Associations with PTSD, shame, and partners’ responses. J. Interpers. Violence.

[80] de Montigny Gauthier L., Vaillancourt-Morel M.P., Rellini A., Godbout N., Charbonneau-Lefebvre V., Desjardins F., Bergeron S. (2019). The risk of telling: A dyadic perspective on romantic partners’ responses to child sexual abuse disclosure and their associations with sexual and relationship satisfaction. J. Marital Fam. Ther..

[81] Campbell S.B., Renshaw K.D. (2012). Distress in spouses of Vietnam veterans: Associations with communication about deployment experiences. J. Fam. Psychol..

[82] Lynch S.H., Shuster G., Lobo M.L. (2018). The family caregiver experience–examining the positive and negative aspects of compassion satisfaction and compassion fatigue as caregiving outcomes. Aging Ment. Health.

[83] Klarić M., Frančišković T., Pernar M., Nemčić Moro I., Milićević R., Černi Obrdalj E., Salčin Satriano A. (2010). Caregiver burden and burnout in partners of war veterans with post-traumatic stress disorder. Coll. Antropol..

[84] Whealin J.M., Yoneda A.C., Nelson D., Hilmes T.S., Kawasaki M.M., Yan O.H. (2017). A culturally adapted family intervention for rural Pacific Island veterans with PTSD. Psychol. Serv..

[85] Dekel R., Goldblatt H., Keidar M., Solomon Z., Polliack M. (2005). Being a wife of a veteran with posttraumatic stress disorder. Fam. Relat..

[86] Gérain P., Zech E. (2022). A harmful care: The association of informal caregiver burnout with depression, subjective health, and violence. J. Interpers. Violence.

[87] Goldstein C., Glass N.E. (2020). Interpersonal Violence: A Review of Elder Abuse. Curr. Trauma Rep..

[88] Caska C.M., Smith T.W., Renshaw K.D., Allen S.N., Uchino B.N., Birmingham W., Carlisle M. (2014). Posttraumatic stress disorder and responses to couple conflict: Implications for cardiovascular risk. Health Psychol..

[89] Maloney L.J. (1988). Post traumatic stresses on women partners of Vietnam veterans. Smith Coll. Stud. Soc. Work.

[90] Brewin C.R., Andrews B., Valentine J.D. (2000). Meta-analysis of risk factors for posttraumatic stress disorder in trauma-exposed adults. J. Consult. Clin. Psychol..

